# Hypoxia-induced invadopodia formation: a role for β-PIX

**DOI:** 10.1098/rsob.120159

**Published:** 2013-06

**Authors:** Nur Fariesha Md Hashim, Nicole S. Nicholas, Anna E. Dart, Serafim Kiriakidis, Ewa Paleolog, Claire M. Wells

**Affiliations:** 1 Division of Cancer Studies, King's College London, London SE1 1UL, UK; 2 Kennedy Institute of Rheumatology, University of Oxford, London W6 8LH, UK; 3 Faculty of Medicine and Health Sciences, Department of Biomedical Science, University Putra Malaysia, Malaysia

**Keywords:** hypoxia, invadopodia, β-PIX

## Abstract

During tumour progression, oxygen tension in the microenvironment surrounding tumour cells is reduced, resulting in hypoxia. It is well established that cancer cells resist the negative effects of hypoxia by inducing angiogenesis predominantly via the activity of transcription factor hypoxia-inducible factor-1 (HIF-1). However, more recently HIF-1α has also been linked to increased invasive potential, although the molecular mechanisms remain to be defined. Invasive cancer cells are thought to employ membrane protrusions, termed invadopodia, to achieve matrix degradation. While many invadopodia components have been identified, signalling pathways that link extracellular stimuli to invadopodia formation remain largely unknown. Indeed, the relationship between invadopodia formation and HIF-1α has not been explored. We now report that HIF-1α is a driver of invadopodia formation. Furthermore, we have identified an important, direct and novel link between the Rho family activator β-PIX, HIF-1α and invadopodia formation. Indeed, we find that β-PIX expression is essential for invadopodia formation. In conclusion, we identify a new HIF-1α mechanistic pathway and suggest that β-PIX is a novel downstream signalling mediator during invadopodia formation.

## 2. Introduction

The development of metastatic disease is a major driver of mortality in patients [1]. During tumour progression, oxygen tension in the microenvironment surrounding the tumour cells is reduced, resulting in intratumoral hypoxia [2,3]. Cancer cells are able to resist the negative effects of hypoxia predominantly via stabilizing and thus promoting the activity of transcription factor hypoxia-inducible factor-1 (HIF-1) [4]. It is well established that HIF-1α can promote angiogenesis [5]; however, tumour hypoxia is also linked to increased metastatic potential in several types of cancers [6–8].

While promotion of angiogenesis has been extensively studied, the molecular mechanisms coupling hypoxia to metastasis are less well understood. In recent years, evidence has started to emerge that HIF-1α activity might also influence the intrinsic invasive behaviour of the cancer cell. HIF-1α activity has been implicated in the induction of epithelial to mesenchymal transition [9], and downregulation of HIF-1α expression has been reported to suppress glioma, colon cancer and melanoma cell migration *in vitro* [10–12]. Expression analysis has revealed that a number of genes which regulate cancer invasion, such as CXCR4, pyruvate dehydrogenase kinase 1 matrix metalloproteinase 2, urokinase plasminogen activator receptor and fibronectin 1 are regulated by HIF-1α [11,12]. Indeed, it has recently been shown that HIF-1α can modulate cell migration via induction of JMY expression [13], and that HIF-1α may regulate c-Met and RhoE expression levels [14,15].

To escape the confines of the primary tumour, cancer cells invade the surrounding stroma and migrate towards local vasculature. Such tissue invasion requires cells to degrade extracellular matrix. Cancer cells are thought to employ actin-rich membrane protrusions, termed invadopodia, to achieve matrix degradation [16,17]. Invadopodia provide protrusive force coupled with the delivery of matrix-degrading metalloproteases to drive cell invasion, and are characterized *in vitro* as having an actin-rich central core associated with cortactin [17]. Invadopodia are similar in architecture and function to the well-characterized invasive protrusions used by haematopoietic cells, termed podosomes [17]. In recent years, invadopodia have been identified in cancer cells from many different tissue types, including breast, and have been studied extensively *in vitro* [18]. Moreover, there is now emerging evidence that these processes are also employed *in vivo* [19]. Many proteins have been implicated in regulating the formation of invadopodia, including members of the Rho family GTPases Rac and Cdc42 [20], p-21-activated kinases [21], and proteins also associated with other forms of cell–substratum adhesion such as paxillin and N-WASP [22,23]. It has been shown that invadopodia activity can be increased by exposure to growth factor signalling, and it has been suggested that exposing cancer cells to a hypoxic environment can lead to increased invasive activity [24], although this study did not link invadopodia formation to the level of HIF-1α expression. Moreover, how invadopodia-forming activity is induced by extracellular signals and coordinated within the cell remains to be fully understood. To better understand specifically how increased levels of HIF-1α contribute to invasive behaviour, we have investigated the formation of invadopodia in breast cancer cells under hypoxic conditions using chemical induction, environmental and overexpression approaches.

## 3. Results and discussion

### 3.1. HIF-1α expression increases invadopodia formation

**Figure 1.**
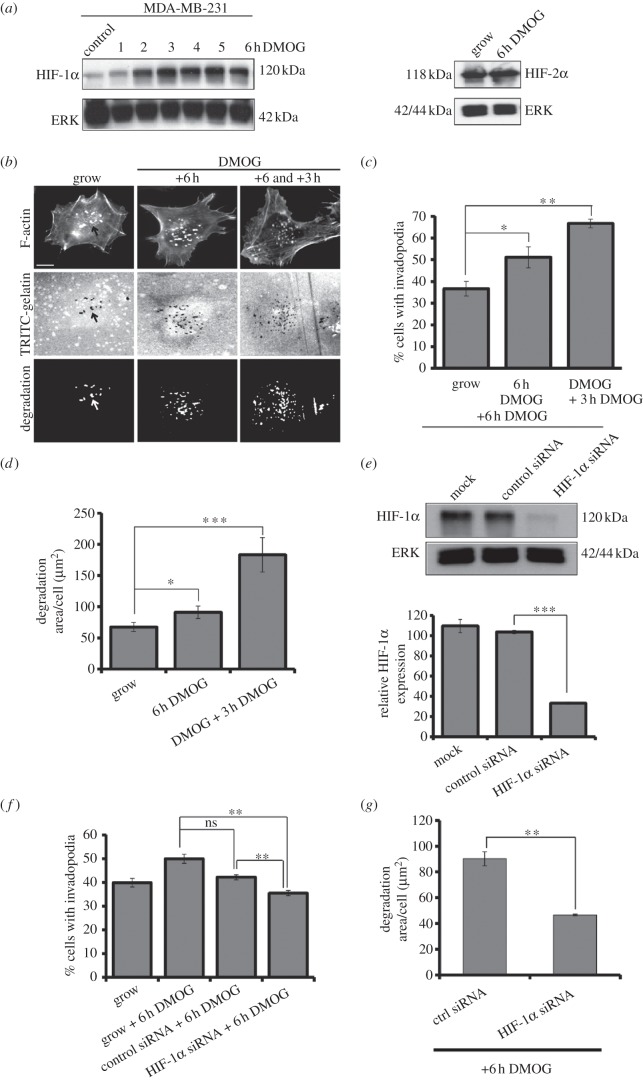
DMOG treatment increases invadopodia formation. (*a*) MDA-MB-231 cells were stimulated with DMOG. Cell lysates were probed for HIF-1α expression and HIF-2α expression with total ERK used as a control. These results are representative of three independent experiments. (*b*) Cells were stimulated with DMOG for 6 h then seeded on TRITC-gelatin-coated coverslips for 3 h, fixed and stained for F-actin. (*c*) Cells were scored for the presence of actin spots with underlying gelatin degradation. (*d*) The gelatin degradation assay results were analysed using ImageJ software. (*e*) MDA-MB-231 cells were transfected with siRNA oligos as indicated. Cells were then treated with DMOG for 6 h. Cell lysates were probed for levels of HIF-1α and ERK. Relative expression of HIF-1α was measured using densitometric analysis. (*f*) HIF-1α KD cells were seeded on gelatin-coated coverslips. Cells were scored as 1C. (*g*) The gelatin degradation assay results were analysed using ImageJ software. In all cases, the results are mean ± s.e.m. of 30 cells from each experimental condition over three separate experiments. Statistical significance was calculated using unpaired, two-tailed *t-*test. *p < 0.05, **p < 0.005 and ***p < 0.0005. Error bars represent s.e.m. Scale bar = 10 µm.

An established protocol to mimic hypoxia-induced stabilization of HIF-1α in cancer cells  is to use di-methyl-oxaloyl-glycine (DMOG), a cell-permeable pan-hydroxylase inhibitor [25]. Addition of DMOG to the cell culture medium quickly stabilized HIF-1α levels (figure 1*a*) with no change in HIF-2α expression levels (figure 1*a*). Moreover, we found a significant increase in the number of cells exhibiting invadopodia-forming activity in the presence of DMOG (figure 1*b,c*). Furthermore, this result was replicated in a second cancer cell line: A375 melanoma cells (see the electronic supplementary material, figure S1*c–e*). While we were surprised to detect changes in invadopodia formation following 6 h in the presence of DMOG, this is not altogether unexpected as others have shown changes in protein levels following 3 h addition of DMOG [26]. Interestingly, we found that sustained presence of DMOG throughout the assay further elevated the response (figure 1*b,c*). Not only did a higher percentage of the cell population exhibit invadopodia-forming activity, but we also noted that there appeared to be an increase in gelatin degradation in those cells that had been exposed to DMOG. These observations were confirmed by quantifying the level of degradation per cell (figure 1*d*; electronic supplementary material, figure S1*f*). Thus, DMOG not only increases levels of invadopodia activity, but also stimulates increased degradative activity in those cells.

These results suggested that HIF-1α directly influences the behaviour of the cancer cells. DMOG is a pan-hydroxylase inhibitor that can not only inhibit the HIF prolyl hydroxylases, but also other hydroxylases [25]; and DMOG has been associated with non-HIF-1α-associated events [27]. To validate the role of HIF-1α in driving an increase in invadopodia-forming activity, we specifically reduced the level of HIF-1α expression in DMOG-maintained cells without affecting the level of related protein HIF-2α (figure 1*e*; electronic supplementary material, figure S1*b*). In the background of reduced HIF-1α expression, we found that DMOG was no longer able to stimulate an increased level of invadopodia formation in these cells (figure 1*f*). Interestingly, degradation analysis of those cells in the HIF-1α population that were able to initiate some invadopodia-forming activity in the presence of DMOG revealed a significant reduction in degradative ability. The significant decrease in gelatin degradation further emphasizes the specific importance of HIF-1α expression in the generation of efficient invasive capacity.

**Figure 2.**
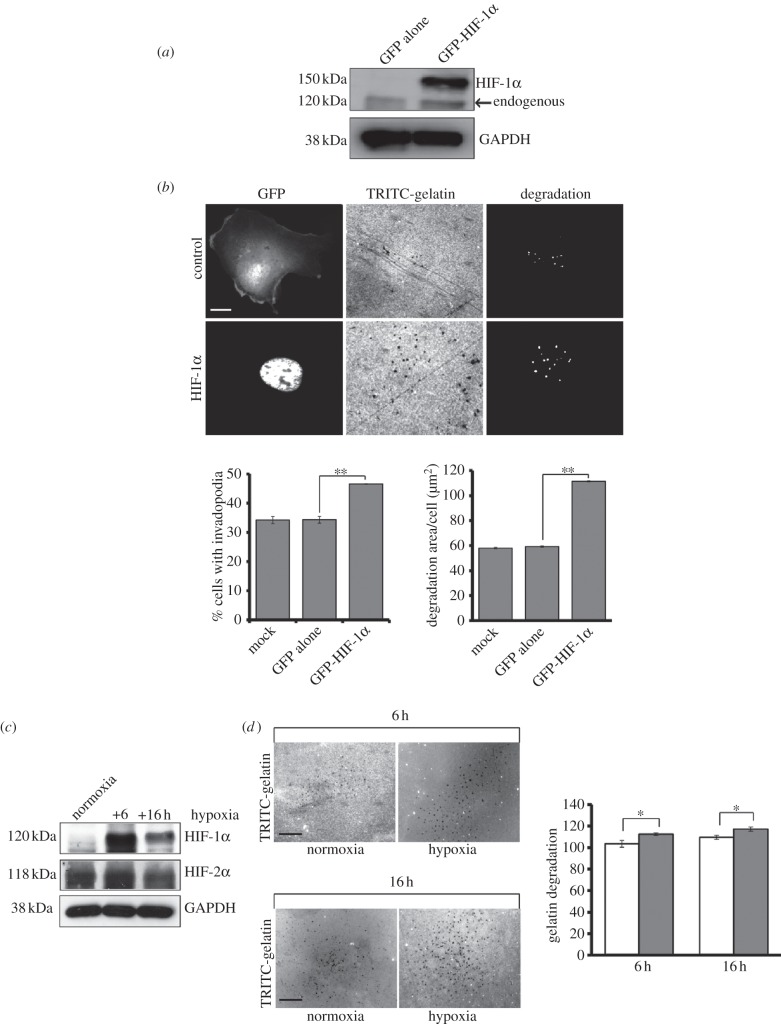
Specific HIF-1α overexpression induces invadopodia formation. (*a*) Lysates from GFP-HIF-1α-transfected cells were probed for HIF-1α expression and GAPDH. (*b*) GFP-HIF-1α-transfected cells were seeded on gelatin coverslips, fixed and stained for F-actin. Cells were scored for the presence of GFP-HIF-1α expression in the nucleus and corresponding actin puncta that colocalize with area of degradation on the gelatin. A gelatin degradation assay was performed using ImageJ software. (*c*) Cells were incubated in normoxia or hypoxia incubators for 6 and 16 h. Cell lysates were probed for levels of HIF-1α and re-probed for HIF-2α. GAPDH was used as a loading control. (*d*) Cells were seeded on gelatin-coated coverslips and incubated in normoxia (open bars) or hypoxia (filled bars) incubator for 6/16 h. The area of degradation on the gelatin was measured using ImageJ software. The results shown are mean grey value ± s.e.m. of 30 fields of view from each experimental condition over three separate experiments. Statistical significance was calculated using Student's *t*-test; *p < 0.05, **p < 0.005, scale bar = 10 µm.

Our findings support a specific role for HIF-1α in invadopodia formation. We next tested whether overexpression of HIF-1α alone could increase invadopodia-forming potential. We generated a green fluorescent protein (GFP)-tagged HIF-1α construct which was able to overcome endogenous HIF-1α degradation pathways and deliver a significant increase in HIF-1α expression, without altering levels of HIF-2α (see the electronic supplementary material, figure S1*c*; figure 2*a*) as well as correctly localizing to the nucleus (see the electronic supplementary material, figure S2*a*). GFP-HIF-1α functioned normally in cells, as demonstrated by the increase in VEGF expression detected in cells expressing GFP-HIF-1α (see the electronic supplementary material, figure S2*b*). Satisfied that GFP-HIF-1α is functionally active, we measured the invadopodia-forming ability of GFP-HIF-1α-expressing cells. GFP-HIF-1α-expressing cells exhibited a significant increase in invadopodia formation compared with control cells and increased levels of gelatin degradation (figure 2*b*).

To complement our induced HIF-1α studies, we used a hypoxia incubator. Incubation of cells under hypoxic conditions stabilized HIF-1α levels, with no effect on HIF-2α levels (figure 2*c*). However, HIF-1α is rapidly degraded when cells are removed from the hypoxia incubator (data not shown). Thus, we were unable to remove cells from the incubator and seed onto gelatin coverslips without losing HIF-1α expression. Instead, by seeding the cells onto gelatin coverslips first and then keeping the cells under hypoxic conditions, we were able to show that increased HIF-1α expression (figure 2*c*) resulted in a concomitant increase in matrix degradation (figure 2*d*) when compared with cells seeded onto gelatin coverslips and maintained in normoxic conditions. Taken together these results strongly support a role for hypoxia, via HIF-1α activity, in driving invasive potential.

### 3.2. HIF-1α regulates expression of cytoskeletal-associated proteins

**Figure 3.**
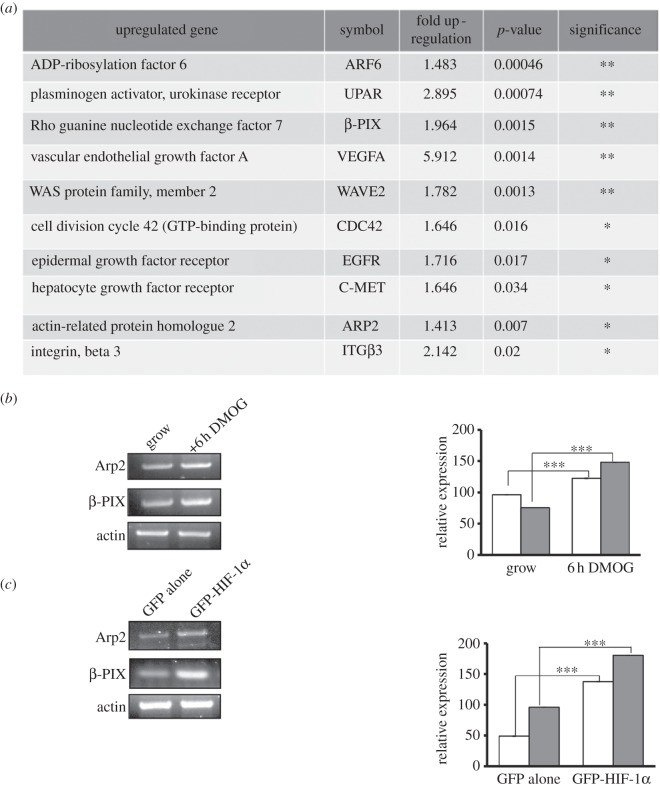
HIF-1α modulates gene expression. (*a*) Total cellular RNA was isolated from DMOG-treated cells, template cDNAs synthesized and used in a Human Cell Motility PCR Array. Data were analysed using a web-based analysis tool (SABiosciences website). The top 10 hits are displayed here. (*b,c*) Total cellular RNA was isolated from DMOG-treated or HIF-1α overexpressing cells. Template cDNAs were synthesized and semi-quantitative (RT)-PCR was performed using Arp2 (filled bars) and β-PIX-specific (open bars) primers. Relative expression was calculated using densitometric analysis. The results shown are mean ± s.e.m. from each experimental condition over three separate experiments. Statistical significance was calculated using Student's *t*-test; *p < 0.05, **p < 0.005.

We reasoned that the effect of HIF-1α expression may result from changes in invadopodia-specific cytoskeletal-related gene expression. To test this hypothesis, we performed PCR array analysis of gene expression following addition of DMOG. As expected, VEGFA and c-Met levels were significantly increased upon the addition of DMOG, but we also found increased expression of a number of cytoskeletal-related genes, including Cdc42, Arp2 and  β-PIX (figure 3*a*; electronic supplementary material, table S1). This is the first time that Arp2 and β-PIX have been identified as potentially HIF-1α regulated. Cdc42 and Arp2 are known regulators of invadopodia formation [23], and we validated HIF-1α-induced Arp2 levels (see the electronic supplementary material, figure S2*c,d*). In contrast, while β-PIX has been associated with increased formation of actin columns in podosome-forming smooth muscle cells, it has not been directly linked to invadopodia formation or matrix degradation. β-PIX is a guanine nucleotide exchange factor for Rac and Cdc42 that has been implicated in the regulation of cell adhesion and cell migration [28,29]. β-PIX is found in a complex with PAK, a protein thought to regulate invadopodia formation [21], thus we decided to investigate the link between β-PIX, HIF-1α and invadopodia formation.

### 3.3. A role for β-PIX in invadopodia formation

**Figure 4.**
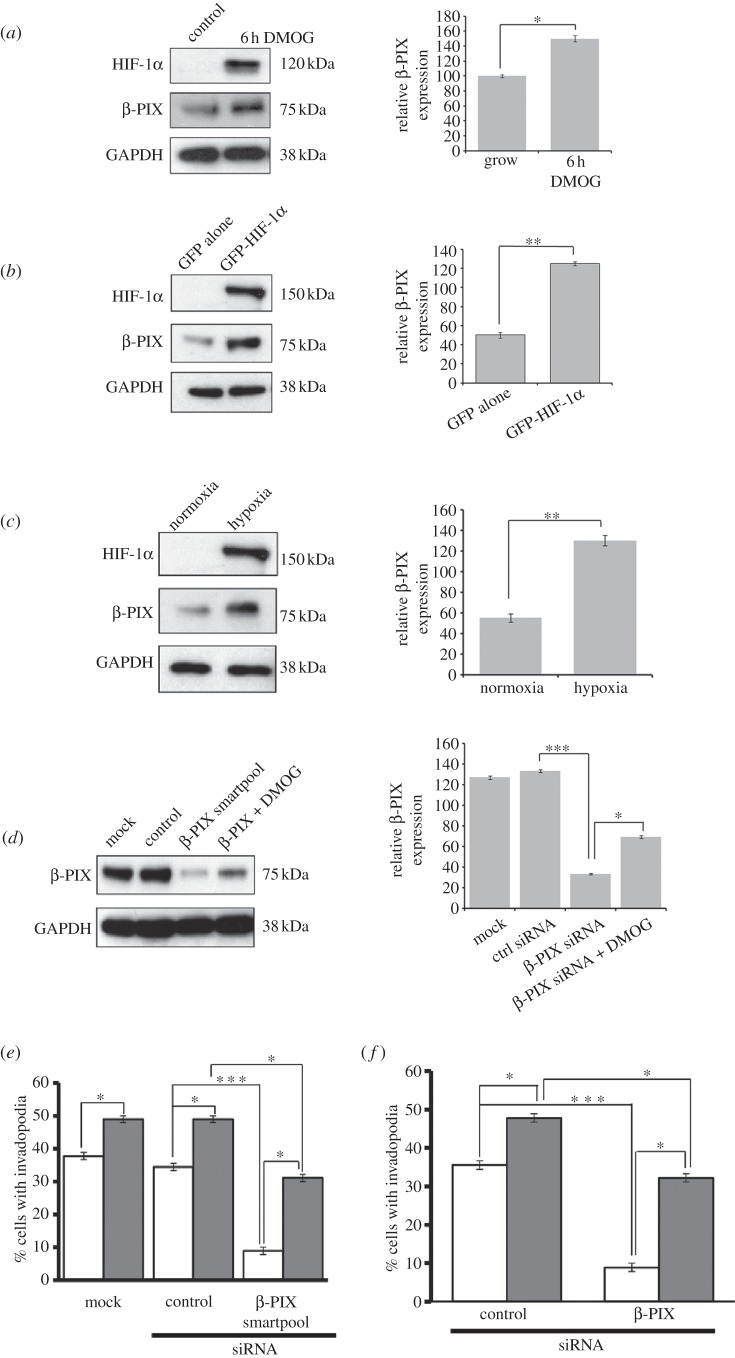
β-PIX is required for invadopodia formation. (*a*) DMOG stimulated, (*b*) GFP-HIF-1α overexpressing cells and (*c*) hypoxic cell lysates were probed for expression of HIF-1α, β-PIX and GAPDH as a loading control. (*d*) MDA-MB-231cells were transfected with mock, control and β-PIX siRNAs for 24 h, ±DMOG for 6 h. Cell lysates were probed for levels of β-PIX and GAPDH as a loading control. (*e*) MDA-MB-231 cells transfected with mock, control and β-PIX siRNAs for 24 h, ±DMOG (+, filled bars; –, open bars) for 6 h were seeded on gelatin-coated coverslips. Cells were scored as figure 1*c*. (*f*) Cells expressing GFP alone (open bars) or GFP-HIF-1α (filled bars) were transfected with β-PIX siRNAs for 24 h then seeded on gelatin-coated coverslips. Cells were scored as figure 1*c*. In all cases, relative expression of β-PIX was measured using densitometric analysis over three separate experiments. In all cases, the results shown are mean±s.e.m. of 30 cells from each experimental condition over three separate experiments. Statistical significance was calculated using Student's *t*-test; *p < 0.05, ***p < 0.0005.

**Figure 5.**
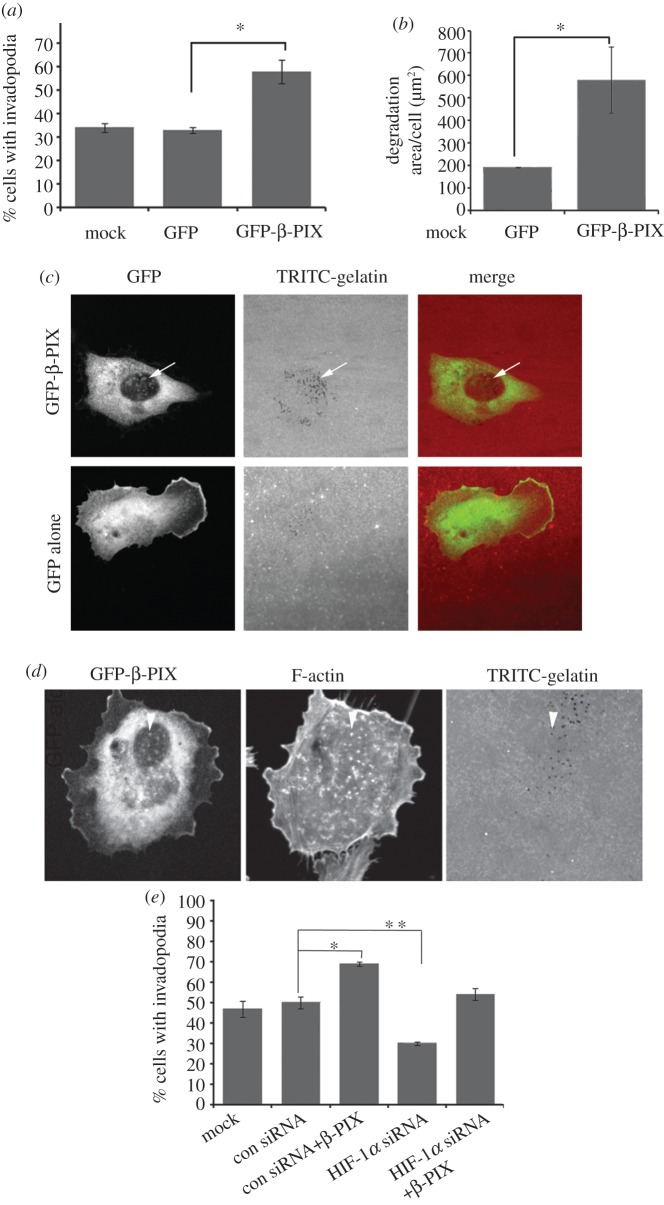
β-PIX can drive invadopodia formation. (*a*) MDA-MB-231 cells mock transfected, expressing GFP alone or expressing GFP-β-PIX were seeded on TRITC-gelatin-coated coverslips for 3 h, fixed and stained for F-actin cells were scored for the presence of actin spots with underlying gelatin degradation. (*b*) The gelatin degradation assay results from cells described in (*a*) were analysed using ImageJ software. (*c*) Representative images of GFP alone and GFP-β-PIX-expressing cells indicating the level of gelatin degradation and the partial co-localization of β-PIX with areas of degradation (arrow). (*d*) Representative image of a MDA-MB-231 cell expressing GFP-β-PIX and treated with DMOG for 6 h prior to seeding on a gelatin-coated coverslip. Arrowhead indicates co-localization of β-PIX, actin puncta and gelatin degradation. (*e*) MDA-MB-231 cells were mock transfected, or transfected with con or HIF-1α-specific siRNA oligos; some cells were then further transfected with GFP-β-PIX as indicated. All cells were then treated with DMOG for 6 h, then seeded on TRITC-gelatin-coated coverslips for 3 h, fixed and stained for F-actin. Cells were scored for the presence of actin spots with underlying gelatin degradation.

We have been able to confirm at the RNA (figure 3*b,c*) and protein level (figure 4*a,b*) that increased HIF-1α levels lead to increased levels of β-PIX using both DMOG and expression of GFP-HIF-1α. Moreover, we have identified a putative HIF-1α response element in the β-PIX gene (see the electronic supplementary material, figure S2*e*). In addition, we detect an increase in β-PIX expression when cells are switched to a hypoxic environment (figure 4*c*). We next speculated that invadopodia formation induced by HIF-1α might be mediated at least in part through the activity of β-PIX. Strikingly, we found that a reduction in β-PIX expression (figure 4*d*) leads to a dramatic decrease in invadopodia formation even under basal conditions (figure 4*e*), and that this reduction in activity could also be detected in a second cancer cell line (see the electronic supplementary material, figure S3). This would suggest a fundamental requirement for β-PIX activity. This role for β-PIX would seem to be distinct from a previously identified role in adhesion disassembly [28], and suggests that the activity of β-PIX is both spatially and temporally regulated. This fundamental requirement for β-PIX activity is further emphasized by the finding that, in a background of β-PIX knockdown, neither DMOG treatment nor GFP-HIF-1α overexpression is able to induce invadopodia formation in cells to control levels (figure 4*e,f*). There is still an induction in invadopodia formation in the β-PIX knockdown background; however, this activity could also be attributed to the fact that β-PIX levels recover in the presence of DMOG (see right lane in figure 4*d*). These data suggest that HIF-1α elevates β-PIX expression, because β-PIX is a fundamental driver of invadopodia formation, rather than that β-PIX only functions in a HIF-1α transcriptional pathway. Indeed, it is probably that HIF-1α may act through multiple cytoskeletal pathways to induce invadopodia formation, a hypothesis supported by our array studies. Nevertheless, our studies point to an essential requirement for β-PIX activity. Given these novel findings, we then tested whether overexpression of β-PIX might also influence invadopodia-forming activity in these cells. We found a striking increase in the level of invadopodia formation in cells expressing GFP-β-PIX compared with GFP alone (figure 5*a*). This was further reflected in the level of gelatin degradation detected in  β-PIX-overexpressing cells (figure 5*b*). Indeed, cells expressing GFP-β-PIX exhibited the highest level of gelatin degradation recorded in this study, again suggesting that HIF-1α upregulates β-PIX expression as a means to directly increase invasive potential. A role for β-PIX in invadopodia formation has not been previously reported, but PIX family members have been implicated in the regulation of podosome formation [30,31], and β-PIX was putatively localized to actin puncta at the cell periphery of those smooth muscle cells. We also found that GFP-β-PIX was localized to the cell periphery, but could also be detected in a cloud around the central core of the cell, often concentrated over the area of predominant gelatin degradation (see the electronic supplementary material, figure S3*c*; figure 5*c*). Moreover, in some cells it was possible to localize GFP-β-PIX puncta to areas of gelatin degradation, and this was more obvious in cells that had been treated with DMOG (figure 5*c* arrow and figure 5*d* arrowhead).

β-PIX is normally associated with focal adhesion dynamics via its interaction with PAK and GIT1, most recently linked to the disassembly of such adhesions [32]. GIT1 is an ADP-ribosylation factor GTPase-activating protein (ARFGAP), which specifically regulates ARF6 [33]. Interestingly, ARF6 has been implicated in the formation of invadopodia downstream of hepatocyte growth factor (HGF) [34], and ARF6 was transcriptionally upregulated by stabilization of HIF-1α in our array (figure 3*a*). Given this close relationship, it might be reasonable to speculate that the requirement for β-PIX in invadopodia formation could also implicate GIT1, ARF6 and/or PAK family proteins**.** However, our data suggest that in the context of invadopodia dynamics β-PIX is a driver of assembly rather than a driver of disassembly. It will be interesting to discover whether the same β-PIX-associated protein interactions function in both scenarios. To further validate our hypothesis that β-PIX is a potent driver of invadopodia formation, we expressed GFP-β-PIX in DMOG-treated cells in the presence of HIF-1α-specific siRNA oligos. Consistent with our previous results, reduced HIF-1α expression prevented cells from responding to DMOG treatment. However, in cells where HIF-1α levels were reduced but β-PIX levels were elevated by overexpression of GFP-β-PIX, we found a level of invadopodia-forming activity comparable with control cells (figure 5*e*). Taken together, these data clearly establish an important, direct and novel link between HIF-1α, invadopodia formation and the Rho family activator β-PIX.

## 4. Material and methods

### 4.1. Cell culture

A375M cells were grown in 10 per cent FBS, 90 per cent DMEM F-12, 15 mM Hepes, supplemented with 10 U ml^−1^ penicillin/10 µg ml^−1^ streptomycin and maintained in a humidified 95 per cent O_2_, 5 per cent CO_2_ incubator at 37°C. HEK-293 cells and MDA-MB-231 cells were grown in 10 per cent FBS, DMEM + Glutamax-1, supplemented with 10 U ml^−1^ penicillin/10 µg ml^−1^ streptomycin and maintained in a humidified 95 per cent O_2_, 5 per cent CO_2_ incubator at 37°C. Cells were treated with DMOG purchased from Sigma at a final concentration of 0.5 mM for times indicated. Cells were transfected using Lipofectamine 2000 according to the manufacturer's protocol. In the case of hypoxic stimulations, cells were incubated at 37°C in a Galaxy R (New Brunswick Scientific) CO_2_ incubator under an atmosphere of 1 per cent O_2_ and 5 per cent CO_2_ for the specified amount of time. For invadopodia analysis, adherent cells were incubated with non-enzymatic cell dissociation solution (Gibco). Dislodged cells were then re-suspended in culture medium. A total of 2 × 10^4^ cells ml^−1^ were seeded onto each gelatin-coated coverslip. Cells were then incubated at 37°C for 3 h before paraformaldehyde fixation.

### 4.2. Reagents

Anti-HIF-1α, anti-p44/42 MAPK (Erk1/2) anti-Cool1/β-PIX were purchased from Cell Signaling Technology (Danvers, MA). Anti-Arp2 was purchased from Abcam (Cambridge, MA). Anti-EPAS1/HIF-2α and anti-GAPDH were purchased from Santa Cruz Biotechnology (Santa Cruz, CA). Anti-β-actin was purchased from Sigma. HRP-conjugated anti-mouse and anti-rabbit were purchased from Dako. Gelatin was from porcine skin type A, Rhodamine B isothiocyanate. Human full-length HIF-1α cDNA clone was purchased from Origene Technologies. GFP-HIF-1α (pDEST201 plasmid) expression vector was generated using Gateway-Cloning System (Invitrogen). Mouse GFP-β-PIX was a kind gift from Dr. Jeff Peterson, Fox Chase Cancer Centre, Philadelphia, USA.

### 4.3. Preparation of gelatin-coated coverslips

Briefly, gelatin was dissolved in 61 mM NaCl, 50 mM NaB4 ph9.3 at 37°C for 1 h, Rhodamine (Sigma) added to the gelatin buffer and mixed for 4 h in the dark at room temperature. The rhodamine-conjugated gelatin (TRITC-gelatin) was then dialysed against PBS. Dialysed TRITC-gelatin was stored with sucrose. Sterile acid-washed coverslips were coated with pre-warmed TRITC-gelatin solution incubated in 0.5 per cent  glutaraldehyde in the dark and then washed with sterile PBS. The coverslips were quenched with 5 mg ml^–1^ sodium borohydride–PBS at room temperature in the dark followed by washing with sterile PBS. The coverslips were then sterilized in 70 per cent ethanol, dried and quenched with DMEM for 1 h at 37°C.

### 4.4. RNAi

MDA-MB-231 cells were seeded at a density of 4 × 10^4^ cell ml^−1^ in 2 ml complete growth medium, allowed to adhere for 24 h and transfected with siRNA oligos using HiPerfect (Qiagen) according to the manufacturer's protocol. Cells were transfected with 75 nM small interfering (si) RNA targeting HIF-1α (Ambion, catalogue no. 4392420) or 75 nM non-specific siRNA (Qiagen) as control. For β-PIX siRNA, 75 nM of a pool of four siRNA duplexes each designed to target human β-PIX (siGENOME SMARTpool) was transfected for 24 h.

### 4.5. PCR analysis

Total cellular RNA was isolated with an RNeasy Plus Mini Kit (Qiagen). Template cDNAs were synthesized with Superscript III (Invitrogen). Semi-quantitative reverse-transcriptase (RT)-PCR was performed with Red-Taq PCR mix (Sigma). See the electronic supplementary material for primer sequences used. A Human Cell Motility PCR Array was purchased from SABiosciences (a Qiagen company). cDNA template was mixed with the ready-to-use PCR master mix and loaded onto an ABI 7700 HT sequence detector (Applied Biosystems, Foster City, CA). Data were analysed using a web-based analysis tool (SABiosciences).

### 4.6. Immunoblotting and immunofluorescence

Cells were lysed and immunoblotted as previously described [35]. The cells were fixed with 4 per cent paraformaldehyde and permeabilized in 0.2 per cent triton X-100/PBS followed by PBS washes. Cells were then incubated with Alexa Fluor 488 phalloidin for 1 h. Cells were then washed twice with PBS. Cells were imaged on an Olympus IX71 fluorescence microscope and a LSM 510 Zeiss confocal microscope. For percentage of cells with invadopodia quantification, individual cells were imaged at high magnification (maximum three cells per field of view) and scored for the presence of punctate gelatin degradation under the cell area as determined by F-actin staining.

### 4.7. Gelatin degradation analysis

ImageJ software (gelatin degradation plug-in by kind gift of Prof. Laura Machesky) was used to measure area of matrix degradation per invadopodia-forming cell. Cell and gelatin images were converted to 8 bits copy prior to combining all cell and gelatin images in separate stack (cell images in one stack and gelatin images on another stack). These stacks were then opened in ImageJ software to measure total degradation area per cell. Data were then multiplied by pixel area (square micrometres) to get real degradation area.

## Supplementary Material

FigureS1_T

## Supplementary Material

Supplemental Methods
