# Supplementary material for: Hypoxia-induced invadopodia formation: a role for β-PIX
Source: Open Biol. 2013 Jun;3(6):120159. doi: 10.1098/rsob.120159 (PMC3718326; doi:10.1098/rsob.120159)
Supplement: FigureS1_T [file rsob120159-s1.pdf]

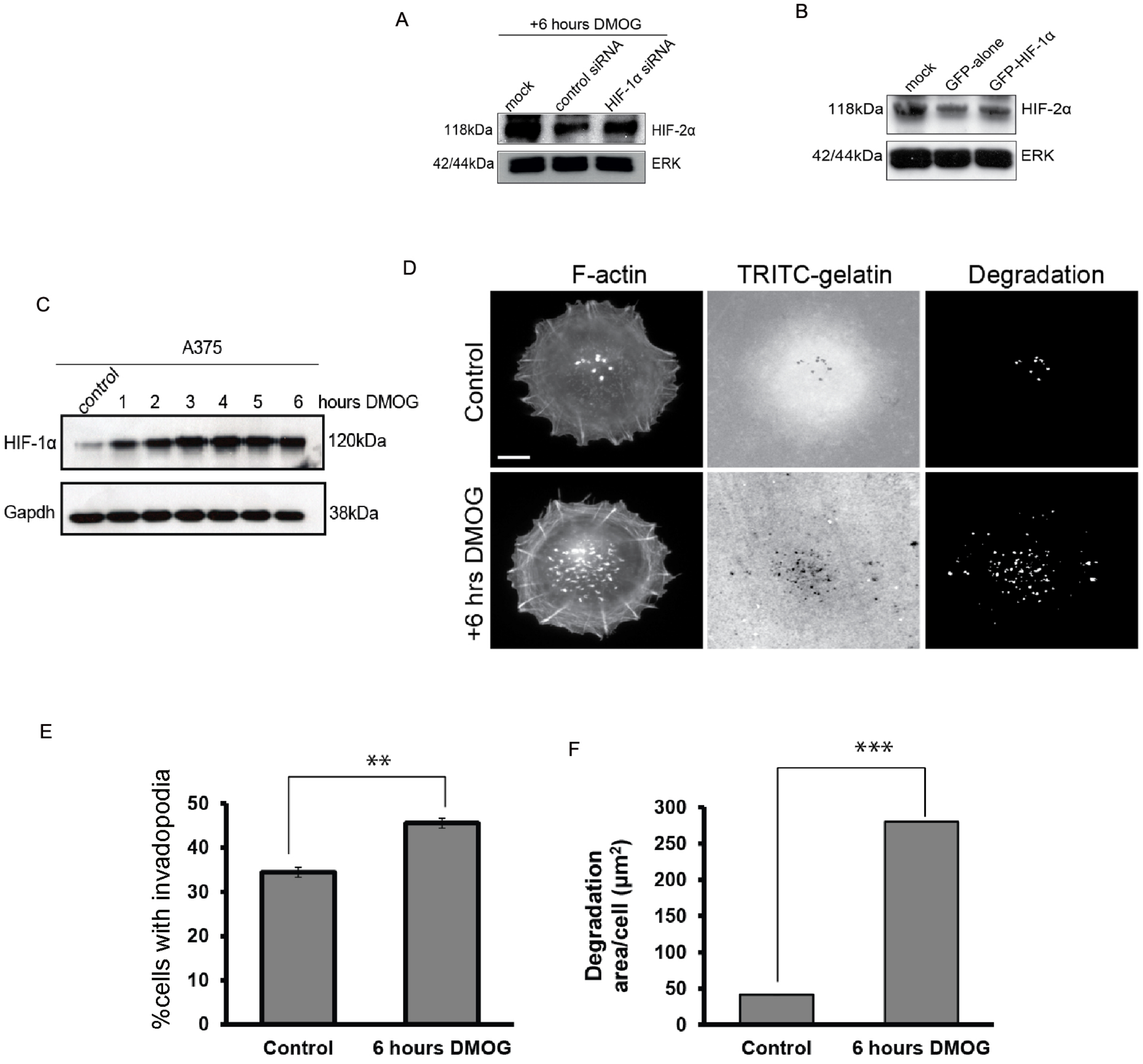

Figure S1. Control for HIF-2a and A375 DMOG treatment A) Cells were transfected with mock, control and HIF-1α siRNAs for 48 hours, followed by stimulation with DMOG for 6 hours. B) Cells were transfected with GFP-HIF-1α. Lysates were probed for levels of HIF-2α and ERK as a loading control. These results are representative of three independent experiments. Relates to Figure 1 and Figure 2. C) A375 cells were stimulated with DMOG for up to 6 hours. lysates were probed for levels of HIF-1α and GAPDH as a loading control. These results are representative of three independent experiments. D) A375 cells were stimulated with DMOG for 6 hours, re-seeded on gelatin-coated coverslips and allowed to adhere for 3 hours. E) Cells were then fixed and stained for F-actin A375 cells were scored for the presence of actin puncta that colocalize with area of degradation on the gelatin and the mean percentage of cells with invadopodia formation calculated. Scale bar=10μm. F) Gelatin degradation assay was performed in all three conditions. The results shown are mean ±SEM of 30 cells from each experimental condition over three separate experiments. Statistical significance compared with control was calculated using Student's *t*-test; *, $P<0.05$. **, $P<0.005$. Relates to Figure 1

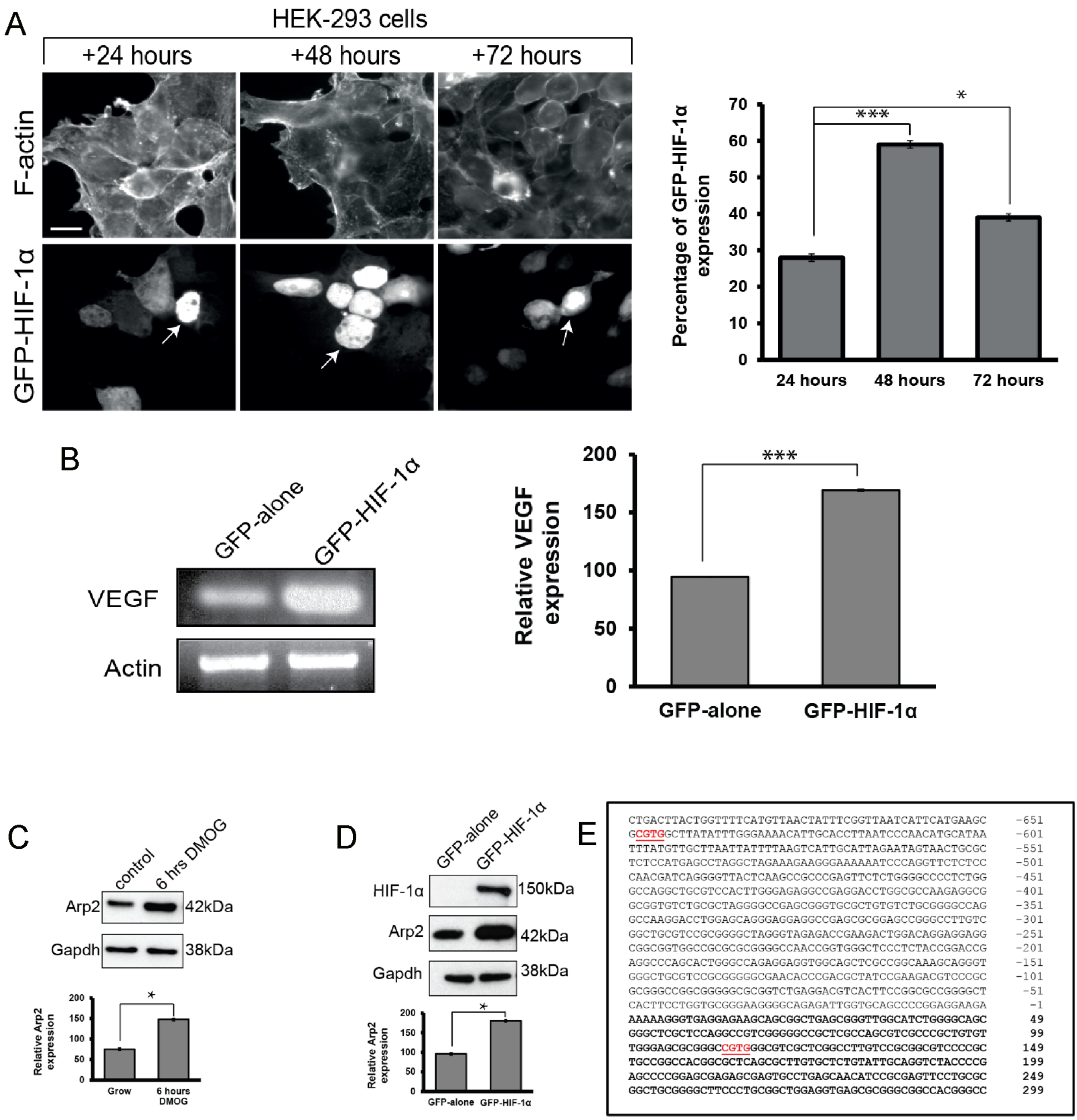

Figure S2A) HEK-293T cells were transfected with GFP-HIF-1α , fixed at 24, 48 and 72 hours following transfections and stained for F-actin. Cells were scored for the presence of GFP-HIF-1α expressions in the nucleus at every indicated time points (arrow). Scale bar=10μm. B) RT-PCR of MDA-MB-231 cells transfected with GFP-control and GFP-HIF-1α using VEGF specific primers. Relative expression of VEGF was measured using densitometric analysis. All the results shown are mean ±SEM from each experimental condition over three separate experiments. Statistical significance was calculated using Student's t-test; *, P<0.05. **, P<0.005. Relates to Figure 1. C) and D) lysates from DMOG treated or HIF-1[illegible] overexpressing cells were probed for Arp2 and GAPDH as a loading control. Relative expression of protein level was calculated using densitometric analysis. E) Two putative hypoxia-response elements (HREs) are located in the promoter sequence of β-PIX. The putative HREs are shaded in red and underlined. Bold shading indicates start of the first exon.

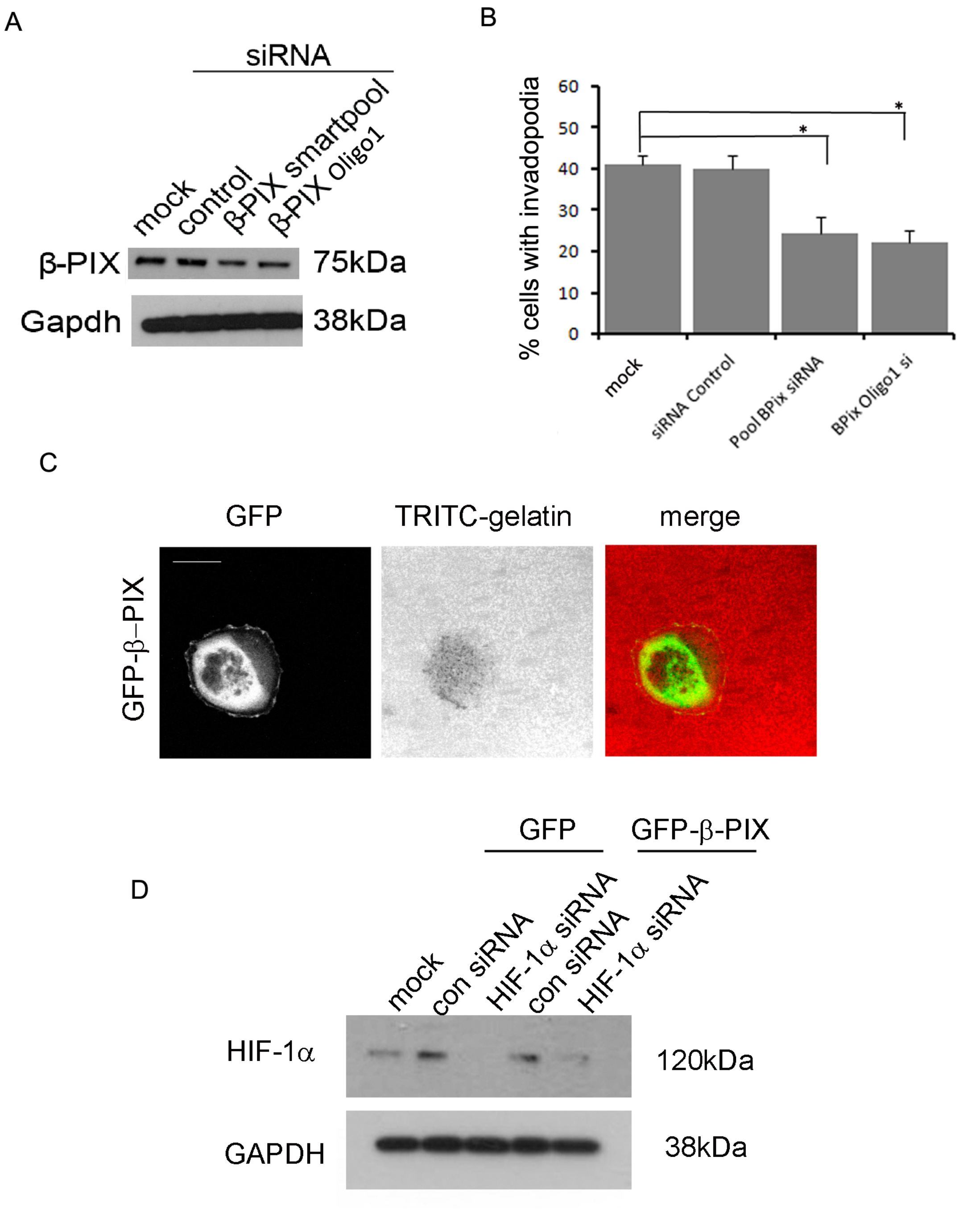

Figure S3. A) A375 cells were treated with control or β-PIX specific si RNA oligos. whole cell lysates were probed for β-PIX expression and GAPDH was used as a loading control. B) A375 cells treated as in (A) were seeded onto gelatin coated coverslips, incubated for 3 hours then fixed and stained for F-actin. Cells were scored as previously described (see materials and methods for details).data represents mean +/- S.E.M over three seperate experiments C) example of β-PIX -GFP localisation in MDA-MB-231 cells and gelatin matrix degradation. D) MDA-MB231 cells were transfected mock, con or HIF-1a si Oligos as indicated. Cells were then transfected with GFP-alone or GFP-β-PIX cells were incubated for 24 hours and then lysed and probed for the level of HIF-1α knockdown or seeded onto gelatin coated coverslips - see Figure 5E
Bar = 10um * = $p < 0.05$

Table S1:
Full PCR Array results. Total RNA was extracted from MDA-MB-231 cells treated/non-treated with DMOG for 6 hours. RNA was converted into cDNA which was used with the master-mix provided in the Human Cell Motility PCR Array kit (Qiagen). Data was processed using the programme provided by the SA Biosciences website. Results of two independent experiments are shown.

Symbol	Well	AVG ΔC_t (Ct(GOI) - Ave Ct (HKG))		$2^{\wedge}-\Delta C_t$		Fold Change	T-TEST	Fold Up- or Down-Regulation
		Test Sample	Control Sample	Test Sample	Control Sample	Test Sample /Control Sample	p value	Test Sample /Control Sample
ACTN1	A01	1.76	2.04	2.9E-01	2.4E-01	1.21	0.276371	1.21
ACTN3	A02	11.91	12.61	2.6E-04	1.6E-04	1.62	0.009594	1.62
ACTN4	A03	1.85	2.14	2.8E-01	2.3E-01	1.22	0.263954	1.22
ACTR2	A04	1.31	1.81	4.0E-01	2.9E-01	1.41	0.006918	1.41
ACTR3	A05	1.34	1.47	3.9E-01	3.6E-01	1.09	0.496009	1.09
AKT1	A06	3.93	3.98	6.6E-02	6.3E-02	1.03	0.695883	1.03
ARF6	A07	1.54	2.11	3.4E-01	2.3E-01	1.48	0.000461	1.48
ARHGDIA	A08	2.45	2.23	1.8E-01	2.1E-01	0.85	0.004745	-1.17
ARHGEF7	A09	3.86	4.84	6.9E-02	3.5E-02	1.96	0.001467	1.96
BAIAP2	A10	6.91	7.75	8.3E-03	4.6E-03	1.79	0.001493	1.79
BCAR1	A11	5.46	5.36	2.3E-02	2.4E-02	0.93	0.000397	-1.08
CAPN1	A12	6.07	6.05	1.5E-02	1.5E-02	0.99	0.995925	-1.01
CAPN2	B01	0.11	0.40	9.3E-01	7.6E-01	1.22	0.016200	1.22
CAV1	B02	0.21	0.66	8.7E-01	6.3E-01	1.37	0.050492	1.37
CDC42	B03	1.08	1.80	4.7E-01	2.9E-01	1.65	0.016355	1.65
CFL1	B04	-1.59	-1.87	3.0E+00	3.7E+00	0.82	0.045061	-1.22
CRK	B05	2.94	3.15	1.3E-01	1.1E-01	1.16	0.187203	1.16
CSF1	B06	1.83	1.75	2.8E-01	3.0E-01	0.95	0.786278	-1.05
CTTN	B07	5.90	6.00	1.7E-02	1.6E-02	1.07	0.437352	1.07
DIAPH1	B08	4.68	5.13	3.9E-02	2.9E-02	1.36	0.185391	1.36
DPP4	B09	10.74	11.32	5.8E-04	3.9E-04	1.49	0.037688	1.49
EGF	B10	7.73	8.62	4.7E-03	2.5E-03	1.86	0.002203	1.86
EGFR	B11	2.81	3.59	1.4E-01	8.3E-02	1.72	0.016685	1.72
ENAH	B12	10.46	10.35	7.1E-04	7.7E-04	0.93	0.718879	-1.08
EZR	C01	0.98	1.22	5.1E-01	4.3E-01	1.18	0.001460	1.18
FAP	C02	8.55	9.12	2.7E-03	1.8E-03	1.49	0.087958	1.49
FGF2	C03	7.24	7.17	6.6E-03	7.0E-03	0.95	0.486860	-1.05
HGF	C04	12.67	13.03	1.5E-04	1.2E-04	1.28	0.369591	1.28
IGF1	C05	11.92	12.16	2.6E-04	2.2E-04	1.18	0.243220	1.18
IGF1R	C06	4.48	4.64	4.5E-02	4.0E-02	1.12	0.476319	1.12
ILK	C07	3.69	3.61	7.8E-02	8.2E-02	0.95	0.676750	-1.06
ITGA4	C08	5.08	5.45	3.0E-02	2.3E-02	1.29	0.281935	1.29
ITGB1	C09	-0.26	-0.07	1.2E+00	1.0E+00	1.14	0.093323	1.14
ITGB2	C10	6.09	6.16	1.5E-02	1.4E-02	1.05	0.934516	1.05
ITGB3	C11	5.06	6.16	3.0E-02	1.4E-02	2.14	0.016352	2.14
LIMK1	C12	4.61	4.42	4.1E-02	4.7E-02	0.87	0.492810	-1.15
MAPK1	D01	3.69	3.58	7.7E-02	8.4E-02	0.92	0.738430	-1.08
MET	D02	2.14	2.86	2.3E-01	1.4E-01	1.65	0.033910	1.65
MMP14	D03	2.59	2.36	1.7E-01	2.0E-01	0.85	0.016178	-1.17
MMP2	D04	7.03	7.50	7.7E-03	5.5E-03	1.39	0.233786	1.39
MMP9	D05	5.89	5.87	1.7E-02	1.7E-02	0.99	0.810845	-1.01
MSN	D06	-0.13	0.22	1.1E+00	8.6E-01	1.27	0.114553	1.27
MYH10	D07	4.35	4.50	4.9E-02	4.4E-02	1.10	0.697104	1.10
MYH9	D08	1.06	1.58	4.8E-01	3.3E-01	1.43	0.037418	1.43
MYL9	D09	3.31	2.13	1.0E-01	2.3E-01	0.44	0.000309	-2.28
MYLK	D10	5.70	4.66	1.9E-02	4.0E-02	0.49	0.067095	-2.05
PAK1	D11	4.94	3.90	3.3E-02	6.7E-02	0.49	0.203755	-2.05
PAK4	D12	5.00	5.26	3.1E-02	2.6E-02	1.20	0.305651	1.20
PFN1	E01	-1.44	-1.50	2.7E+00	2.8E+00	0.97	0.636501	-1.04
PIK3CA	E02	4.00	3.83	6.2E-02	7.0E-02	0.89	0.562075	-1.13
PLAUR	E03	0.41	1.95	7.5E-01	2.6E-01	2.90	0.000737	2.90
PLCG1	E04	4.99	4.68	3.2E-02	3.9E-02	0.81	0.251637	-1.24
PLD1	E05	6.16	6.04	1.4E-02	1.5E-02	0.92	0.611809	-1.09
PRKCA	E06	4.31	4.35	5.0E-02	4.9E-02	1.02	0.956182	1.02
PTEN	E07	2.51	2.58	1.8E-01	1.7E-01	1.05	0.973415	1.05
PTK2	E08	2.79	3.31	1.4E-01	1.0E-01	1.43	0.299814	1.43
PTK2B	E09	7.44	7.24	5.8E-03	6.6E-03	0.87	0.555507	-1.15
PTPN1	E10	3.63	2.75	8.1E-02	1.5E-01	0.54	0.036235	-1.84
PXN	E11	2.15	2.39	2.3E-01	1.9E-01	1.18	0.197938	1.18
RAC1	E12	0.13	0.03	9.1E-01	9.8E-01	0.94	0.453375	-1.07
RAC2	F01	3.51	3.24	8.8E-02	1.1E-01	0.83	0.514015	-1.20
RASA1	F02	2.57	2.89	1.7E-01	1.3E-01	1.25	0.646520	1.25
RDX	F03	3.90	3.79	6.7E-02	7.3E-02	0.92	0.683627	-1.08
RHO	F04	10.44	11.32	7.2E-04	3.9E-04	1.84	0.019255	1.84
RHOA	F05	-0.01	-0.08	1.0E+00	1.1E+00	0.95	0.178589	-1.05
RHOB	F06	2.67	2.69	1.6E-01	1.6E-01	1.01	0.981426	1.01
RHOC	F07	1.42	1.45	3.7E-01	3.7E-01	1.02	0.795575	1.02
RND3	F08	2.68	2.28	1.6E-01	2.1E-01	0.75	0.251663	-1.33
ROCK1	F09	6.26	6.13	1.3E-02	1.4E-02	0.91	0.566919	-1.10
SH3PXD2A	F10	5.32	5.25	2.5E-02	2.6E-02	0.95	0.778565	-1.05
SRC	F11	6.19	6.13	1.4E-02	1.4E-02	0.96	0.610721	-1.04
STAT3	F12	2.71	2.70	1.5E-01	1.5E-01	0.99	0.853448	-1.01
SVIL	G01	4.86	4.67	3.4E-02	3.9E-02	0.87	0.575728	-1.15
TGFB1	G02	2.30	3.17	2.0E-01	1.1E-01	1.82	0.051426	1.82
TIMP2	G03	1.75	1.69	3.0E-01	3.1E-01	0.96	0.734263	-1.04
TLN1	G04	2.35	2.67	2.0E-01	1.6E-01	1.25	0.258196	1.25
VASP	G05	5.54	6.55	2.2E-02	1.1E-02	2.02	0.007259	2.02
VCL	G06	2.73	2.48	1.5E-01	1.8E-01	0.84	0.413964	-1.19
VEGFA	G07	-0.03	2.54	1.0E+00	1.7E-01	5.91	0.001374	5.91
VIM	G08	-2.75	-3.07	6.7E+00	8.4E+00	0.80	0.107016	-1.25
WASF1	G09	4.29	4.60	5.1E-02	4.1E-02	1.23	0.577541	1.23
WASF2	G10	2.45	3.28	1.8E-01	1.0E-01	1.78	0.001317	1.78
WASL	G11	7.08	7.53	7.4E-03	5.4E-03	1.36	0.298065	1.36
WIPF1	G12	5.58	11.96	2.1E-02	2.5E-04	83.21	0.035535	83.21
B2M	H01	-0.74	-0.60	1.7E+00	1.5E+00	1.10	0.294318	1.10
HPRT1	H02	3.58	3.84	8.4E-02	7.0E-02	1.20	0.176251	1.20
RPL13A	H03	-0.20	-0.20	1.1E+00	1.2E+00	1.00	0.839547	-1.00
GAPDH	H04	-2.68	-2.86	6.4E+00	7.3E+00	0.88	0.198335	-1.13
ACTB	H05	-2.64	-3.04	6.2E+00	8.2E+00	0.76	0.006593	-1.32
