# Supplementary material for: Hypoxia-induced invadopodia formation: a role for β-PIX
Source: Open Biol. 2013 Jun;3(6):120159. doi: 10.1098/rsob.120159 (PMC3718326; doi:10.1098/rsob.120159)
Supplement: Supplemental Methods [file rsob120159-s2.doc]

**Supplemental Methods**

**Primer sequences**

β-PIX forward (5’-ATGAATTCCGCCGAGCAAACC-3’),

β-PIX reverse (5’-TTAGTTCTGGTGAGAGATATA-3’),

Arp2 forward (5’-ATGGACAGCCAGGGCAGGAAG-3’),

Arp2 reverse (5’-TTATCGAACAGTCACACCAAG-3’),

VEGF forward (5’-CTTGCCTTGCTGCTCTACCT-3’),

VEGF reverse (5’-CTGCATGGTGATGTTGGACT-3’),

Actin forward (5’- ATGTGCAAGGCCGGCTTCG-3’) and

Actin reverse (5’- TAGAAGCATTTGCGGTGGA-3’).
